# Comparative Analysis of the Efficacy of Various Retreatment File Systems in the Removal of Gutta-Percha in Retreatment Cases and Time Taken During the Procedure: An In Vitro Cone Beam CT Study

**DOI:** 10.7759/cureus.55551

**Published:** 2024-03-05

**Authors:** Kapilesh B Singh, Mohammad Salman Akhtar, Renuka Nagar, Abhinay Agarwal, Saleem Azhar, Vikas Singh

**Affiliations:** 1 Conservative Dentistry and Endodontics, SMBT Dental College & Hospital, Mumbai, IND; 2 Conservative Dentistry and Endodontics, Teerthanker Mahaveer Dental College & Research Centre, Moradabad, IND; 3 Public Health Dentistry, Teerthanker Mahaveer Dental College & Research Centre, Moradabad, IND

**Keywords:** cone beam computed tomography (cbct), niti files, root canal treatment, gutta percha, retreatment files system

## Abstract

Background

Removing gutta-percha manually can be a challenging task, especially when addressing densely packed root-filling material, particularly in cases where resin-based sealers are employed for obturation. The use of nickel-titanium (NiTi) rotary instruments not only effectively shapes the root canal but also efficiently removes the endodontic filling from the curved canal during retreatment. Hence, incorporating rotary NiTi instruments in retreatment cases can alleviate fatigue for both patients and operators.

Objectives

This study aims to compare the efficacy of Neo-Endo retreatment files, R-Endo retreatment files, and K and H files in the removal of endodontic filling material. Additionally, the remnants of gutta-percha in root canals are evaluated using cone beam computed tomography (CBCT).

Materials and methods

A total of 60 extracted first maxillary molar teeth were selected for this study. Canal preparation was conducted using the step-back method up to an apical size of 40 K-file. The obturation process involved the use of gutta-percha points and AH Plus sealer in a lateral compaction technique. Post-operative CBCT scans were taken. The samples were randomly divided according to the retreatment files used: group I included Neo-Endo retreatment files, group II included R-Endo retreatment files, and group III included conventional K-files and Hedstroem files (H-Files). The retreatment procedure was considered complete when the last instrument easily reached the working-length range and was physically clean. A stopwatch was used to record the time taken by each file to remove the obturating material. T1 represented the total time (including irrigation and change of file) required to reach the apex, while T2 indicated the complete removal of materials from the canal with the last instrument. The overall time recorded (TT) was calculated as T1 + T2. The removal process was analyzed with CBCT scans.

Results

The Neo-Endo retreatment files removed the filling materials better and more quickly than the other files.

Conclusions

Despite the presence of residual filling material in all samples, the Neo-Endo retreatment files left the least amount of residual filling material and achieved the shortest completion time.

## Introduction

Despite the continuous advancements in technology and materials, endodontic treatment failures remain a common phenomenon [1]. Most of these failures are attributed to the presence of microflora in the root canal system, primarily due to insufficient debridement [2]. Due to inadequate cleaning, untreated canals, insufficient filling, or coronal or apical microleakage, bacteria present in the canal system may persist or reappear, leading to disease [3, 4]. The main purpose of non-surgical endodontic retreatment is to restore healthy periapical tissue [1]. The objective of this procedure is to thoroughly eliminate the filling material from the root canal system. This allows the irrigant and intracanal medicaments to adequately penetrate the intricate structure of the root canal and ensure proper filling.

However, the manual removal of gutta-percha can be a tedious process, more so when the root-filling material is compacted and resin-based sealers are used for obturation [3]. In such cases, the nickel-titanium (NiTi) rotary instrument proves highly effective in shaping the root canal and removing the endodontic filling from the curved canal during retreatment [5]. Hence, using rotary NiTi instruments in retreatment cases can alleviate patient and operator fatigue [3].

Nevertheless, all retreatment techniques leave residues on the walls of the canal after re-instrumentation [6]. Studies have demonstrated that no retreatment techniques can completely clean the walls of the root canal, especially in the apical region [4].

The Neo-Endo retreatment system is specifically designed for efficient and effective removal of gutta-percha and resin-based sealers during retreatment procedures. It offers a comprehensive set of instruments tailored for various aspects of retreatment, including coronal, middle, and apical thirds of the root canal. This system is known for its versatility and compatibility with different root canal anatomies, allowing clinicians to achieve thorough cleaning and shaping of the root canal system. On the other hand, the R-Endo files are NiTi rotary instruments designed to facilitate the removal of gutta-percha and other obturation materials from curved root canals. These files feature advanced cutting geometries and flexible properties, enabling precise and efficient removal of filling materials while minimizing the risk of procedural errors or instrument fracture [3].

Various methods have been employed to evaluate the effectiveness of devices and techniques in the removal of obturating materials. One such method is cone beam computed tomography (CBCT), which offers a non-invasive approach to visualizing the detailed morphology of the root canal without the need for tooth destruction or three-dimensional (3D) evaluation of the treatments performed within the root canal system [5].

So far, there are few studies in the literature on the 3D volumetric evaluation of the remaining obturation materials and the efficacy of retreatment files. Currently, there is a notable lack of data regarding the efficacy of Neo-Endo retreatment files, a recently introduced system, in comparison to other rotary retreatment systems currently available. Limited research exists regarding retreatment in ultraconservative access openings utilizing rotary retreatment files. Therefore, this study was undertaken to evaluate and compare the effectiveness of various manual and rotary NiTi instruments in the removal of resin-based root canal obstruction materials during root canal retreatment.

## Materials and methods

Study design

A total of 60 extracted maxillary first molars were collected, adhering to specific inclusion and exclusion criteria. These criteria ensured that all teeth had a standardized palatal root length of 16 mm and fully formed apical foramina. Additionally, these teeth were devoid of any previous endodontic treatment or retreatment, exhibited radiographic evidence of a single canal in the palatal root, and were amenable to the lateral compaction technique of obturation. The ethical approval for the study was taken from the ethical committee of SMBT Dental College and Hospital, Sangamner, Maharashtra, India, with the institutional review board number (IRB) IEC/SMBT/2022/56.

Inclusion and exclusion criteria

The study excluded teeth with severely curved or anatomically irregular root canals, signs of external root resorption, extensive periapical pathology, previous root fractures, or multiple canals in the palatal root from the study. Similarly, teeth with calcified or obliterated root canals, a history of contraindications to irrigants such as sodium hypochlorite (NaOCl) or ethylenediaminetetraacetic acid (EDTA), known allergies to study materials or medications, and a history of radiation therapy to the head and neck were excluded.

Preparation and measurement protocols

The working length of each tooth (16 mm) was measured by inserting a size 10 K-file into the canal until the tip was visible at the apical foramen. Subsequently, 1 mm was subtracted from the working length measurement. A size 40 K-file was used as the master apical file. The final irrigant used was 17% liquid EDTA followed by normal saline flushing. The lateral compaction technique was employed for obturation. Post-obturation restoration was performed using amalgam, and CBCT scans were taken post-operatively. Finally, the obturated teeth were immersed in saline for 14 days to assess the long-term sealing ability, tissue response, and clinical relevance of the endodontic treatment under investigation.

The following three groups (n = 20 each) were formed for the study.

Group I Neo-Endo (Orikam Healthcare India Private Limited, Haryana, India): The file lengths range from 16 mm to 18 mm for N1 (coronal one-third), 16 mm to 18 mm for N2 (middle one-third), and 22 mm to 25 mm for N3 (apical one-third). The Neo-Endo instruments were utilized at 350 rpm with a torque of 1.5 Ncm^2^.

Group II R-Endo (Bensons Surgico, Delhi, India): The Rm stainless steel hand file (4% taper and 17 mm in length) was first used to full working length, followed by rotary instruments Re (taper l2%), R1 (taper 8%), R2 (taper 6%), and R3 (taper 4%) to working length. The R-Endo instruments were operated at 300 rpm with a torque of 1.2 Ncm^2^.

Group III conventional K-Files and H-Files (Welcare Orthodontics, Delhi, India): Hand files were used alternatively until size 40. During instrumentation, the canals were irrigated with NaOCl. The final rinse was performed using EDTA and saline.

**Figure 1:**
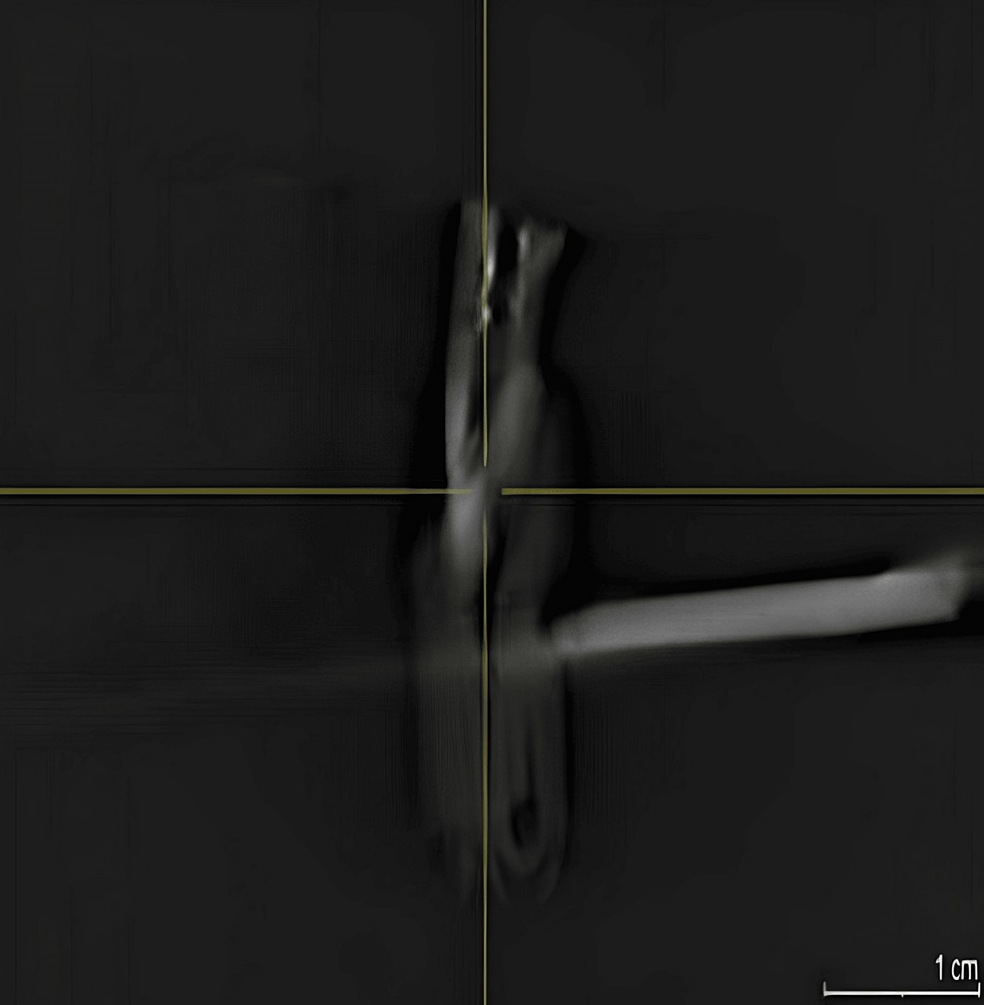
CBCT image after retreatment CBCT, cone beam computed tomography

The completion of the procedure was determined when the last file easily reached the apex and no further reproducing material was present on the instruments. A stopwatch was used to record the time taken for all files to remove the root canal filling. Two time intervals were recorded: T1, which represented the total time (including irrigation and change of file) required to reach the apex, and T2, which represented the total removal of materials with the last file used. The overall time recorded (TT) was calculated as T1 + T2. CBCT with Image I (64-bit) software version 1.47 was employed to test the efficiency of gutta-percha removal (Figure 1).

This software reconstructed the raw data into 3D images of the root canal system, allowing for precise visualization and analysis. Researchers meticulously examined these reconstructed images to assess the efficiency of gutta-percha removal. They identified any remaining gutta-percha within the root canal space and compared these findings to pre-treatment images or standard references to determine the extent of removal.

Statistical analysis

Statistical Package for Social Sciences (SPSS) for Windows Version 22.0 (IBM Corp., Armonk, NY) was used for analysis. Descriptive statistics, including means, standard deviations, and percentages, summarized the characteristics of the study groups and procedural outcomes. Comparative analysis of time intervals, such as T1 (time to reach the apex), T2 (time for total material removal), and TT (total time), was conducted among the three study groups using appropriate statistical tests, including analysis of variance (ANOVA) or non-parametric tests if assumptions of normality were violated. Post hoc tests such as Tukey's honestly significant difference (HSD) test were employed to identify specific pairwise differences in cases of significant overall differences among groups. Statistical significance was set at a predetermined alpha level (e.g., p < 0.05), ensuring reliable interpretation of results.

## Results

Table 1 presents a comparison of different instrumentation techniques used in endodontic treatment.

**Table 1:** Distribution of samples among three groups

Variable	Name of file	Abbreviation	N	%
Group I	Neo-Endo	Neo-Endo	20	33.3
Group II	R-Endo	R-Endo	20	33.3
Group III	Conventional K-files and H-files	K and H files	20	33.3

When comparing the efficacy of hand and rotary retreatment files in terms of the percentage of remaining gutta-percha and time required, ANOVA revealed a significant impact in the groups. Furthermore, within each group, when comparing the percentage of the remaining gutta-percha and time required for the retreatment procedure, the Tukey test demonstrated a significant difference between the groups in terms of the time taken and area required for the retreatment. However, in terms of volume, the difference between the groups was non-significant (Table 2).

**Table 2:** Comparing the effectiveness of two NiTi files and hand files for removing gutta percha and resin-based sealer from palatal root canals’ volume The data are summarized as mean ± standard deviation

Variables			N	Mean	Standard deviation	P-value
							Palatal	Group I	Neo-Endo	20	12.268	0.891	0.03
Group II	R-Endo	20	11.37	0.799									
Group III	K and H files	20	11.605	1.432									
Mesiodistal	Group I	Neo-Endo	20	12.393	0.94	0.005
	Group II	R-Endo	20	11.376	0.833	
	Group III	K and H files	20	11.477	1.311	

Upon post hoc comparison, it was observed that significant differences were seen only between group I and group II, with group I showing the maximum reduction. Similarly, the comparison of removal of gutta-percha point and resin material was conducted using one-way ANOVA for three different file systems. Results indicated a significant difference in the area reduction for the mesiodistal root. Upon post hoc comparison, significant differences were observed between group I and group II as well as between group I and group III, exhibiting the maximum reduction (Table 3).

**Table 3:** Post hoc comparison

Dependent variable			Mean difference	Standard error	P-value
						Palatal	Group I	Group II	0.897	0.341	0.029
Group III	0.662	0.341	0.136				
Group II	Group I	-0.897	0.341	0.029		
	Group III	-0.236	0.341	0.77		
Group III	Group I	-0.662	0.341	0.136		
	Group II	0.236	0.341	0.77		
Mesiodistal	Group I	Group II	1.016	0.331	0.009
		Group III	0.915	0.331	0.021
	Group II	Group I	-1.016	0.331	0.009
		Group III	-0.1	0.331	0.951
	Group III	Group I	-0.915	0.331	0.021
		Group II	0.1	0.331	0.951

The removal of gutta-percha point and resin material was compared using one-way ANOVA for three different file systems. The analysis revealed a significant difference in the reduction of the area for the palatal root (Table 4). 

**Table 4:** Comparing the effectiveness of two NiTi files and hand files for removing gutta percha and resin-based sealer from palatal root canals’ volume The data are summarized as mean ± standard deviation

Variable			N	Mean	Standard deviation	P-value
							Palatal	Group I	Neo-Endo	20	-53.407	6.36	0.409
Group II	R-Endo	20	-51.549	7.965									
Group III	K and H files	20	-50.218	8.106									

Table 5 presents the mean, standard deviation, and p-values for the variables T1 (time taken for removal of gutta -percha point and resin material), T2 (time taken for removal with the last file), and T1+T2 (total time taken). For T1+T2, the mean time taken for group I (Neo-Endo) was 11.454 minutes for group I (Neo-Endo), for group II (R-Endo) was 21.503 minutes for group II (R-Endo), and for group III (K and H files) was 30.208 minutes for group III (K and H files). The ANOVA test yielded a significant p-value of < 0.0001, indicating that there is a statistically significant difference in the time taken for removal among the three groups. For T1, the mean time taken for group I was 3.284 minutes for group I, for group II was 4.515 minutes for group II, and for group III was 7.016 minutes for group III. Again, the ANOVA test yielded a significant p-value of < 0.0001, suggesting a significant difference in the time taken for removal among the groups. Similarly, for T2, the mean time taken for group I was 8.171 minutes for group I, for group II was 16.988 minutes for group II, and for group III was 23.193 minutes for group I. The ANOVA test showed a significant p-value of < 0.0001, indicating a significant difference in the total time taken among the groups. (Table 5).

**Table 5:** Comparing the effectiveness in time taken for two NiTi files and hand files for removing gutta-percha and resin-based sealer from palatal root canals (T2-T1) The data was are summarized as the mean ± SD (standard deviation

Variables	N	Mean	Standard. deviation	95% confidence interval for mean	Minimum	Maximum	P -value
T1	Group I: Neo-Endo	20	3.284	1.229	0.275	2.708	3.859
	Group II: R-Endo	20	4.515	3.132	0.7	3.049	5.98
	Group III: K and H files	20	7.016	2.826	0.632	5.693	8.338
T2	Group I: Neo-Endo	20	8.171	2.691	0.602	6.911	9.43
	Group II: R-Endo	20	16.988	8.264	1.848	13.12	20.856
	Group III: K and H files	20	23.193	8.629	1.929	19.154	27.231
T1+T2	Group I: Neo-Endo	20	11.454	3.307	0.739	9.906	13.002
	Group II: R-Endo	20	21.503	8.519	1.905	17.515	25.49
	Group III: K and H files	20	30.208	8.63	1.93	26.169	34.247

Table 6 presents the post hoc results for pairwise comparisons between the three groups (Neo-Endo, R-Endo, and K and H files) for the variables T1, T2, and T1+T2.

**Table 6:** Post hoc results

Dependent variable	Mean difference	Standard error	P-value
T1	-1.23	0.8	0.28
	1.23	0.8	0.28
	-3.73	0.8	0.001
	-2.5	0.8	0.01
	3.73	0.8	0.001
	2.5	0.8	0.01
T2	-8.81	2.24	0.001
	8.81	2.24	0.01
	-15.02	2.24	0.01
	-6.2	2.24	0.02
	15.02	2.24	0.001
	6.2	2.24	0.02
T1+T2	-10.04	2.29	0.01
	10.04	2.29	0.01
	-18.75	2.29	0.01
	-8.7	2.29	0.01
	18.75	2.29	0.01
	8.7	2.29	0.01

For T1, significant differences were observed between group I and group III (p = 0.001), indicating that the time taken for removal was significantly lower in group I (Neo-Endo) compared to group III (K and H files). For T2, significant differences were found between all pairs of groups: group I vs.versus group II (p = 0.001), group I versusvs. group III (p = 0.01), and group II versusvs. group III (p = 0.02). For T1+T2, significant differences were observed between all pairs of groups: group I versusvs. group II (p = 0.01), group I versusvs. group III (p = 0.01), and group II versusvs. group III (p = 0.01).

## Discussion

The primary objective of root canal retreatment is to remove any contaminated root canal obturating material and correct any previous mishaps. During this process, the practitioner removes the filling material to uncover any potential areas where pulp remnants or microbes may have contributed to the treatment failure. By doing so, irritants can thoroughly clean all the nooks and corners of the root space, thereby attaining disinfection and apical patency [6-8].

To facilitate the treatment, a solvent is utilized to ease the penetration of the retreatment file into the obturating material. However, it is important to note that the solvent can leave a hard layer of gutta-percha stuck to the dentinal walls [9]. Extended immersion periods may result in the degradation of resin-based sealers, potentially compromising their sealing ability and facilitating bacterial leakage or reinfection. Additionally, the immersion medium itself can influence the chemical and mechanical properties of obturation materials and root canal dentin [10].

NiTi instruments plasticize gutta -percha due to frictional heat; hence, the instrument quickly reaches the apex. Thus, this study found Ni-Ti rotary instruments to be more effective in gutta-percha removal. This may be attributed to the design of these instruments. R-Endo instruments are machined on round blanks. The files have three equally spaced cutting edges without any radial lands or active tips. This design helps in the removal of gutta -percha from the canal. Additionally, these instruments have a tip diameter of 0.25 mm with different tapers, resulting in enhanced rigidity, especially in 4% taper files [11].

The Neo-Endo retreatment files have a positive rake angle and high fracture resistance, which improves cutting efficiency, flexibility, and resistance to cyclic fatigue, and they are used in a circumferential filing motion [9].

Various studies have demonstrated good efficacy and decreased preparation time of NiTi rotary instruments compared to hand instruments in retreatment cases [12]. Similarly, this study found that NiTi rotary instruments took less time when compared to hand instruments during retreatment. Mechanical instrumentation plasticizes gutta -percha and reduces resistance to the action of subsequent instrumentation [4]. Consequently, working to the working length with NiTi instruments was easier compared to hand files [13-16]. A recent report revealed that in the case of two dimensions (2D), radiographs failed to represent real cleanliness [11].

In accordance with our study, Karamifar et al. concluded that rotary instrumentation was more effective than hand instruments in gutta-percha removal from canal walls. The XP-endo finisher file, with its metallurgy and elliptical rotation movement, proved to be more effective than other files. Its elliptical rotational movement was particularly efficient in reaching inaccessible parts of the canals [15]. According to Colaco et al.and Pai, rotary gutta-percha removal techniques had significantly fewer gutta-percha remnants than manual gutta-percha removal techniques. Their study compared the efficacy of ProTaper universal retreatment files, D-race retreatment files, and H-files, suggesting that the design of ProTaper retreatment files and D-race retreatment files may contribute to their superior performance [13]. However, Madani et al. found that hand files were more effective in removing obturating material during retreatment. This difference may be because of the dissimilar internal anatomies of the tooth samples [2].

Limitations

The study has several limitations that must be acknowledged. First, the use of extracted teeth may not fully replicate clinical conditions. Additionally, variations in instrument design and clinician experience, along with limited sample size, further contribute to the study’s limitations. Moreover, the use of CBCT for evaluation may have limitations in detecting small amounts of residual material. Finally, the in vitro nature of the study may not fully capture clinical complexities. Despite these limitations, the findings of this study suggest that Neo-Endo retreatment files offer promising results for endodontic retreatment cases. However, it is important to note that further research and clinical studies are necessary to validate and extend these findings to real-world scenarios.

## Conclusions

Under the confines of this study, it can be concluded that all instruments leave some residue on root canal walls. On evaluating the total percentage of leftover material, Neo-Endo files exhibited the highest removal rate, followed by R-Endo files and H and K files. Further, the time required for retreatment was also shorter for the Neo-Endo retreatment files in contrast to others.
